# Hospitalized older adult: predictors of functional decline*

**DOI:** 10.1590/1518-8345.3612.3399

**Published:** 2021-01-08

**Authors:** João Paulo de Almeida Tavares, Lisa Alexandra Nogueira Veiga Nunes, Joana Catarina Gonçalves Grácio

**Affiliations:** 1 Universidade de Aveiro, Escola Superior de Saúde, Aveiro, Portugal.; 2 Centro Hospitalar e Universitário de Coimbra, Centro de Responsabilidade Integrado de Psiquiatria, Coimbra, Portugal.; 3 Centro Hospitalar e Universitário de Coimbra, Serviço de Cirurgia Maxilo-Facial e Cirurgia Plástica, Coimbra, Portugal.

**Keywords:** Aged, Health of the Elderly, Nursing, Hospitalization, Logistic Models, Functional Decline, Idoso, Saúde do Idoso, Enfermagem, Hospitalização, Modelos Logísticos, Declínio Funcional, Anciano, Salud del Anciano, Enfermería, Hospitalización, Modelos Logísticos, Deterioro Functional

## Abstract

**Objective:**

to identify the predictors of functional decline in hospitalized individuals aged 70 or over, between: baseline and discharge; discharge and follow-up, and baseline and three-month follow-up.

**Method:**

a prospective cohort study conducted in internal medicine services. A questionnaire was applied (clinical and demographic variables, and predictors of functional decline) at three moments. The predictors were determined using the binary logistic regression model.

**Results:**

the sample included 101 patients, 53.3% female, mean age of 82.47 ± 6.57 years old. The predictors that most contributed to decline in hospitalization were the following: previous hospitalization (OR=1.8), access to social support (OR=4.86), cognitive deficit (OR=6.35), mechanical restraint (OR=7.82), and not having a partner (OR=4.34). Age (OR=1.18) and medical diagnosis (OR=0.10) were the predictors between discharge and follow-up. Being older, *delirium* during hospitalization (OR=5.92), and presenting risk of functional decline (OR=5.53) were predictors of decline between the baseline and follow-up.

**Conclusion:**

the most relevant predictors were age, previous hospitalization, cognitive deficit, restraint, social support, not having a partner, and *delirium*. Carrying out interventions aimed at minimizing the impact of these predictors can be an important contribution in the prevention of functional decline.

## Abstract (pt)

**Objetivo:**

identificar os preditores do declínio funcional em pessoas hospitalizadas com 70 ou mais anos, entre: *baseline* e alta; alta e *follow-up* e *baseline* e *follow-up* de três meses.

**Método:**

estudo de coorte prospectivo realizado em serviços de medicina interna. Aplicado um questionário (variáveis demográficas, clínicas e preditores do declínio funcional) em três momentos. Os preditores foram determinados utilizando o modelo de regressão logística binária.

**Resultados:**

a amostra incluiu 101 pacientes, 53.3% do sexo feminino, idade média de 82,47 ± 6,57 anos. Os preditores que mais contribuíram para o declínio na hospitalização foram: internação prévia (RC=1,8), acesso a apoio social (RC=4,86), déficit cognitivo (RC=6,35), contenção mecânica (RC=7,82) e não ter parceiro(a) (RC=4,34). A idade (RC=1,18) e o diagnóstico médico (RC=0,10) foram os preditores entre a alta e o *follow-up*. Ser mais velho, *delirium* durante a hospitalização (RC=5,92) e ter risco de declínio funcional (RC=5,53), foram preditores de declinio entre a baseline e o *follow-up*.

**Conclusão:**

os preditores mais relevantes foram idade, internação prévia, déficit cognitivo, contenção, apoio social, não ter parceiro(a) e *delirium*. Executar intervenções orientadas para minimizar o impacto destes preditores pode ser um importante contributo na prevenção do declínio funcional.

## Abstract (es)

**Objetivo:**

identificar los predictores del deterioro funcional en personas internadas de al menos 70 años de edad, entre: *baseline* y alta; alta y *follow-up*, y *baseline* y *follow-up* a los tres meses.

**Método:**

estudio de cohorte prospectivo realizado en servicios de Medicina Interna. Se aplicó un cuestionario (variables clínicas y demográficas, y predictores del deterioro funcional) en tres momentos. Los predictores se determinaron por medio del modelo de regresión logística binaria.

**Resultados:**

la muestra incluyó a 101 pacientes, 53,3% del sexo femenino, con una media de edad de 82,47 ± 6,57 años. Los predictores que más contribuyeron al deterioro durante la internación fueron los siguientes: internación anterior (OR=1,8), acceso a apoyo social (OR=4,86), déficit cognitivo (OR=6,35), restricción mecánica del movimiento (OR=7,82) y no tener pareja (OR=4,34). La edad (OR=1,18) y el diagnóstico médico (OR=0,10) fueron los predictores entre el alta y el *follow-up*. Tener edad avanzada, padecer *delirium* durante la internación (OR=5,92), y presentar riesgo de deterioro funcional (OR=5,53) fueron predictores del deterioro entre la *baseline* y el *follow-up*.

**Conclusión:**

los predictores más relevantes fueron edad, internación anterior, déficit cognitivo, restricción del movimiento, apoyo social, no tener pareja y *delirium*. Realizar intervenciones destinadas a minimizar el efecto de estos predictores puede ser un importante aporte para prevenir el deterioro funcional.

## Introduction

Hospitalization results in functional decline (FD) for the older adults (OAs) due to the interaction between the changes in primary aging, the disease, and the hospital practices^(^ 1 ^-^ 2 ^)^. FD is characterized by the inability to engage in necessary or desirable activities in daily life. FD is not only linked to the clinical condition that led to the hospitalization; as such, its recovery is not automatic after the resolution of the medical problem^(^ 3 ^)^.

The literature highlights multiple factors that put hospitalized people at risk of FD. *Delirium* or the presence of cognitive deficit, low performance in activities of daily living at baseline, advanced age, sensory deficits, depression, and multimorbidity are associated with increased risk of FD and decreased likelihood of improvement after hospitalization^(^ 4 ^-^ 7 ^)^. Additionally, previous hospitalizations^(^ 5 ^)^, the iatrogenic effects of the treatment^(^ 8 ^)^, the absence of an informal support network^(^ 9 ^)^, and the number of falls in the last year^(^ 3 ^)^ also accentuate FD. The factors related to the hospital practices that reinforce low mobility and bed rest^(^ 4 ^,^ 10 ^-^ 14 ^)^, polymedication, the use of psychotropic drugs, direct and/or indirect restraint, malnutrition, and the use of medical devices (e.g., use of vesical catheterization)^(^ 3 ^,^ 15 ^-^ 18 ^)^ are also associated with FD. The hospital environment and politics play an equally significant role^(^ 19 ^-^ 20 ^)^.

The patients who are unable to recover after the hospitalization episode may maintain the declining trajectory^(^ 3 ^,^ 21 ^-^ 22 ^)^, so the predictors of FD must be identified early in order to take preventive measures^(^ 2 ^)^. Many times, these predictors reflect the sociocultural system of the health services and of the development of current care. In Portugal, as far as the authors’ knowledge is concerned, there are no studies that have analyzed the predictors of functional decline in hospitalized older adults aged 70 or over. This study had as its objective to identify the predictors of functional decline in hospitalized people aged 70 years old or older, between: baseline and discharge; discharge and follow-up, and baseline and three-month follow-up.

## Method

A prospective cohort study carried out in internal medicine services in a central public university hospital of the central region of Portugal. Two hospitalization units for men and two for women were included. Each service has 33 beds, with a mean monthly occupation of 100%. The research was approved by the Ethics Commission of the hospital (opinion No. 065-14) and the data were collected after clarification of the study and acquisition of the consent term.

The sample was of the consecutive convenience type. The inclusion criteria were the following: individuals aged ≥70 years old with the ability to understand and interpret the questions of the questionnaire (informed consent to the patients or, if not possible, having an informal caregiver who answered the questionnaire). The exclusion criteria were as follows: older adults transferred from the intensive care unit, older adults with terminal or neurodegenerative disease, totally dependent at baseline (maximum score in the Katz Index), and hospitalizations of less than 48 hours.

Calculation of the sample was conducted and, for a moderate effect size (0.5), level of significance (α) of 0.05 and power (β) of 0.8, the number of 102 participants was obtained. The sample included 117 patients in an initial phase, with intention to compensate for possible drop-outs and, at discharge time, it consisted of 101 participants. Due to this fact, the power of the test for the sample obtained was calculated (*post-hoc*), resulting in a power of 0.8.

In order to identify the largest number of predictors of FD, a questionnaire was carried out which included sociodemographic and clinical data, as well as different scales (Table 1).

**Table 1:** Variables, instruments and moments of data collection. Coimbra, Portugal, 2016

Variable	Instrument	Collection moment
Age, gender, level of schooling, marital status, family nucleus	Sociodemographic questionnaire	Admission
Admission date, admission diagnosis(es), multimorbidity, number of previous hospitalizations, hospitalization period, number and type of medications, and therapeutical attitudes	Clinical questionnaire and Charlson Comorbidity Index	Admission
Cognitive decline	Cognitive Decrease Test (6CIT)^(^ 23 ^)^	Admission
Senses (sight and hearing)	Question "can you see well?"; "can you hear well?"	Admission
Affective state	Single item of the Geriatric Depression Scale^(^ 24 ^)^	During hospitalization (3 to 5 days after admission)
Fear of falling	Single item of fear of falling^(^ 25 ^)^	During hospitalization
Falls	Record of falls during hospitalization	Discharge
Pain	Numerical Scale or Qualitative Scale	During hospitalization
*Delirium*	Confusion Assessment Method^(^ 26 ^)^	Admission and during hospitalization
Restraint	Observation grid of physical restriction^(^ 27 ^)^	During hospitalization
Risk of functional decline	ISAR-HP - Portuguese version^(^ 28 ^)^	Admission
Functional capacity of the OA	Katz Index^(^ 29 ^)^	Admission, discharge and follow-up

**Figure 1:**
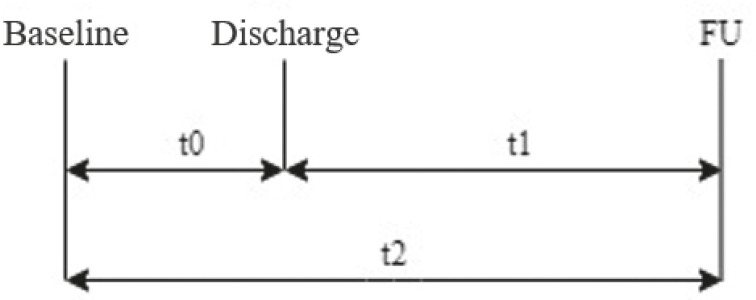
Moments of the assessment of the functionality of the hospitalized older adults

The assessment of the functional capacity was performed with the Katz Index (KI). It is an instrument with six basic activities of daily life (BADL) of dichotomous reply (0=dependent, 1=independent). The older adults and/or caregivers were asked to describe their functional capacity prior to hospitalization (baseline), reporting the last two weeks. The decline in the BADL between baseline and discharge was defined as t0; t1 corresponded to the decline between discharge and follow-up (FU); and t2 reported the decline between baseline and FU (Figure 1). In this study, FD was defined as any decline in one or more points in the KI between the three moments at which the assessment was conducted.

Data collection took place from May to October 2016. In the initial assessment, the patients admitted in the services were analyzed daily in order to identify the ones that presented eligibility criteria. The data were collected by the researchers through: heterofilling of the questionnaire (preferably with the patient or, if not possible, with the informal caregivers, or with the health team: nurses, physicians, and operational assistants), consultation of the clinical diary, of the electronic medical record, and through telephone contact to obtain the follow-up data. A three-month FU was chosen given that some studies reported that, in this period (1^st^ and 3^rd^ months after discharge), a significant number of older adults can recover their functionality^(^ 30 ^-^ 31 ^)^.

In the analysis of the data, techniques of descriptive and inferential statistics were used. In the univariate analysis, the Student’s t test was used (when the normality of the distribution was not verified, Mann-Whitney’s U test was used), as well as ANOVA (when the normality of the distribution was not verified, Kruskal-Wallis H test was used), and the chi-square test with Odds Ratio (OR) (when the estimates of the chi-square test were not verified, Fisher’s exact test was used).

In the multivariate analysis, the binary logistic regression model was used for the three declines under study (t0, t1, and t2). The variables to include in the model were selected in accordance with the following: the number of cases (one predictor for every ten cases), p≤0.15, and predictors with evidence in the literature^(^ 32 ^-^ 34 ^)^. The correlation between the independent variables was analyzed, those with values *≥*0.75 being excluded. The conditional backward selection method was used for the selection of the variables with a predictive value. In the statistical treatment of the data, the IBM SPSS 23 (Statistical Package for the Social Sciences) statistical program was used. A *p*-value <0.05 was considered statistically significant.

## Results

A total of 117 patients were included, of which the following were excluded: 10 due to death, four due to transfers to other services, one due to absence of baseline, and one for hospitalization of less than 48 hours. The final sample consisted of 101 patients. Approximately half of the sample consists of female older adults (53.3%), with a mean age of 82.47 ± 6.57 years old and mostly widowed (49%). More than half presents low schooling (0-2 years) (57.4%), and only 7.9% of the sample has seven or more years of study. They live predominantly in their homes or in family homes (83.2%), and only 17 (16.8%) are institutionalized (2.95 ± 2.79 years). The most prevalent medical admission diagnoses were infectious disease (42.6%), cardiovascular disease (17.8%), and hydroelectrolytic imbalance (12.9%). Twenty-five percent of the patients had been hospitalized in the last year, of which 74.29%, 13.86% and 8.57% were hospitalized once, twice or three times, respectively.

Most of the patients are polymedicated (75.2%) and have four pathologies; the Charlson Comorbidity Index (CCI) (0-8) presented a median of six (4-7) and the Estimated Relative Risk of Death (ERRD) (0-19.37) was 9.23 (4.4-13.4). More than half of the sample reports not seeing well, 54.4% does not hear well, and 71.3% reports weight loss in the last three months. High risk of fall was identified in 48% of the patients, 58.5% of the patients reported being afraid of falling, and four patients fell during their stay in the hospital. Thirty-two point three percent (32.3%) have a high risk of pressure injury. Most of the patients (68%) reported feeling sad or depressed frequently, 58% had no cognitive deficit, nine patients presented *delirium* in the first 48 hours, and eight patients developed *delirium* during hospitalization. The mean length of hospitalization was 9.97 ± 7.02 days.

*Predictors of functional decline:* At t0, in the univariate analysis of the sociodemographic and clinical variables, it was observed that the older adults who declined were significantly more likely to be: male (OR=2.5), 2.7 years old older, without a partner (OR=3.7), and with previous social support; with more hospitalizations within the last year, with a higher risk of falls, higher risk of pressure injury (OR=3.5), cognitive deficit, depression (OR=2.9), *delirium* at admission, and restraint during hospitalization (OR=6.5). Assessed at admission, *delirium* only occurred in patients with decline, reason why it represents an isolated predictive variable (it does not enter into the logistic regression model, for presenting 0 cases in the patients without decline).

The regression model was statistically significant (x^2^(5)=38.85, *p*<0.01). Having been hospitalized in the previous year (OR=1.8), having access to social support prior to hospitalization (OR=4.86), presenting cognitive deficit (OR=6.35), having been subjected to mechanical restraint (OR=7.82), and not having a partner (OR=4.34) are significant predictors of decline during hospitalization. The model explains 52.3% of the variance of functional decline and correctly classified 82.1% of the cases (relation between the true values and those predicted by the model). It was observed that the ROC curve of the predictive model for t0 presented an area of 0.87 (*p*≤0.01) (95% CI: 0.79-0.95), with a standard error of 0.04.

At t1, it was observed that the older adults who declined are likely to be nearly five years old older (median). Decline is also dependent on the medical admission diagnosis, for which there is a positive association between the absence of decline and the diagnosis of infectious disease (n=39; adjusted residue=3.4) and between the presence of decline and the diagnosis of lung disease (n=2; adjusted residue=2.4).

For the clinical variables, the OAs with decline at t1 were more frequently hospitalized in the last year and have higher CCI and ERRD. The place of residence of the OAs, three months after discharge, is dependent on the decline that occurs between discharge and follow-up, with a positive association between decline at t1 and institutionalization at follow-up (n=9; adjusted residue=2.7). The same is true between the absence of decline and home stay (n=51; adjusted residue=2.6). It was observed that the decline is dependent on the ISAR-HP score, so the patients classified as at risk (ISAR-HP≥2) are 11.73 times more likely to have FD in the three months after discharge.

**Table 2:** Sociodemographic and clinical characteristics of the OAs according to functional decline at t0. Coimbra, Portugal, 2016

Clinical characteristics	Patients without decline (n = 74)	Patients with decline (n = 27)	*p*/OR*	Log^†^ regression
				*p*/OR
Age (M^‡^±SD^§^)	81.0 (6.08)	83.69 (6.77)	0.04**	0.76/0.98
Male (%)	20 (42.6)	27 (57.4)	0.03^††^/2.5	0.71/1.32
Without a partner (%)	37 (68.5)	17 (37)	0.01^††^/3.7	0.02/4.34
With social support (t0) (%)	5 (12.5)	14 (31.8)	0.04^††^/0.31	0.04/4.86
Hospitalizations in the last year (*Median*, 1^st^ and 3^rd^)	0 (0 - 0)	0 (0 - 1)	<0.01^¤^	0.03/1.80
Psychoactive drugs	20 (43.5)	34 (61.8)	0.07^††^	0.13/2.77
Does not see well (%)	21 (45.7)	35 (63.6)	0.07^††^	0.32/0.51
Does not hear well (%)	13 (28.3)	23 (41.8)	0.16^††^	0.50/0.56
Fear of falling (%)	20 (47.6)	35 (67.3)	0.05^††^	0.78/1.27
High risk of falls (%)	20 (44.4)	28 (50.9)	0.01^††^	0.23/4.34
High risk of PI^||^(%)	8 (18.2)	24 (43.6)	<0.01^††^/3.5	0.46/1.84
Cognitive decline (%)	12 (26.1)	30 (55.6)	<0.01^††^/3.5	<0.01/6.35
Depression	25 (55.6)	43 (78.6)	0.02^††^	0.97/0.96
*Delirium* at admission (%)	-	9 (16.4)	<0.01^††^/2	-
*Delirium* during hospitalization (%)	2 (4.8)	8 (15.1)	0.18^††^	0.46/2.84
Restraint (%)	6 (14.3)	27 (51.9)	<0.01^††^/6.5	<0.01/7.82
CCF^¶^(*Me*, 1^st^ and 3^rd^ Q)	0.5 (0.22 - 0.56)	0.47 (0.36 - 0.63)	0.30^‡‡^	0.69/1.91
Risk of functional decline (%)	30 (65.2)	47 (85.5)	0.02^††^/3.1	0.84/1.22

* OR = Odds Ratio;

† Log = Logistic;

‡ M = Mean;

§ SD = Standard deviation;

|| PI = Pressure injur;

¶ CCF = Care Centered on Functionality;

** Student's t test;

†† Chi-square test;

‡‡ Mann-Whitney's U test

The regression model was statistically significant (x^2^(2)=28.05, *p*<0*.*01) and showed that age (OR=1.18) and diagnosis (OR=0.10) are significant predictors of functional decline between discharge and follow-up. The model explains 35.3% of the functional decline variance and correctly classified 73.3% of the cases. It was observed that the ROC curve of the predictive model for t1 presented an area of 0.83 (*p*≤ 0.01) (95% CI: 0.74-0.92) with a standard error of 0.05.

At t2, it was verified that the patients who declined were the oldest (approximately five years old older compared to the patients who did not decline), those who were more frequently hospitalized in the last year, those who had CCI and ERRD with higher scores, and those who had longer hospitalizations (two more days – median). They were also those who reported feeling sad or depressed more frequently and had *delirium* episodes during hospitalization. It was observed that functional decline at t2 is dependent on the risk assessment by ISAR-HP, and that the older adults at risk are 10.72 times more likely to have functional decline.

Through the logistic regression (Table 4), whose model is statistically significant (x^2^(3)=25.55, *p*<0*.*01), it was verified that being older, presenting *delirium* during hospitalization (OR=5.92), and presenting risk of decline, identified through ISAR-HP (OR=5.53), are the variables that most predict decline in this period. The model explains 31.8% of the functional decline variance and correctly classified 71.6% of the cases. It was observed that the ROC curve of the predictive model for t2 presented an area of 0.78 (*p*≤ 0.01) (95% CI: 0.69-0.87) with a standard error of 0.05.

**Table 3:** Sociodemographic and clinical characteristics of the OAs according to functional decline at t1. Coimbra, Portugal, 2016

Clinical characteristics	Patients without decline (n = 74)	Patients with decline (n = 27)	*p*/OR*	Log^†^ regression
				*p*/OR
Age (*Median*, 1^st^ and 3^rd^ Q)	81 (76 - 87)	86 (83 - 89)	<0.01^§^	<0.01/1.18
Admission diagnosis (%) Infectious disease	39 (38.6)	4 (4)	<0.01^||^	<0.01/0.10
Number of medications (*Median*, 1^st^ and 3^rd^ Q)	6.5 (3.75 - 10)	8 (6 - 11)	0.11^§^	0.48/1.05
Hospitalizations in the last year (*Median*, 1^st^ and 3^rd^ Q)	0 (0 - 1)	1 (0 - 1)	0.03^§^	0.077/0.92
CCI^‡^ (*Median*, 1^st^ and 3^rd^ Q)	4 (4 - 7)	6 (5 - 8)	0.01^§^	0.21/1.2
Does not hear well (%)	23 (31.1)	13 (48.1)	0.11^¶^	0.92/1.08
Weight loss (%)	49 (66.2)	23 (85.2)	0.06^¶^	0.39/1.84
*Delirium* during hospitalization (%)	5 (7.2)	5 (19.2)	0.13^||^	0.16/3.62
Cognitive decline (%)	28 (37.8)	14 (53.8)	0.15^¶^	0.44/1.593
Risk of functional decline (%)	51 (68.9)	26 (96.3)	<0.01^¶^/11.7	0.14/5.29

* OR = Odds Ratio;

† Log = Logistic;

‡ CCI = Charlson Comorbidity Index;

§ Mann-Whitney's U test;

|| Fisher's test;

¶ Chi-square test

**Table 4:** Sociodemographic and clinical characteristics of the OAs according to functional decline at t2. Coimbra, Portugal, 2016

Clinical characteristics	Patients without decline (n = 74)	Patients with decline (n = 27)	*p*/OR*	Log^†^ regression
				*p*/OR
Age (M^‡^±SD^§^)	80.34 (6.22)	85.77 (5.77)	0.00**	0.04/1.10
With social support (t0) (%)	9 (17.3)	10 (31.3)	0.14^††^	0.43/1.65
Hospitalizations in the last year (%)	0 (0 - 1)	0 (0 - 1)	0.09^‡‡^	0.99/0.99
CCI^||^ (Me, 1^st^ and 3^rd^ Q)	5 (4 - 7)	6 (4 - 7)	0.04^‡‡^	0.29/1.14
ERRD^¶^ (Me, 1^st^ and 3^rd^ Q)	6.38 (4.4 - 13.37)	9.23 (6.38 - 13.37)	0.05^‡‡^	0.87/1.02
Does not hear well (%)	18 (29.5)	18 (45.0)	0.11^††^	0.74/0.83
Cognitive decline (%)	21(34.5)	21 (53.8)	0.06^††^	0.62/1.35
Depression	35 (58)	33 (82.5)	0.02^††^/0.3	0.14/2.35
Delirium during hospitalization (%)	2 (3.6)	8 (20.5)	0.01^§§^	0.06/5.9
Restraint (%)	15 (26.8)	18 (47.4)	0.13^††^	0.88/1.02
Length of hospitalization (Median, 1^st^ and 3^rd^ Q)	7 (5 - 10)	9 (6 - 17.5)	0.05^‡‡^	0.97/0.99
Risk of functional decline (%)	39 (63.9)	38 (95.0)	<0.01^††^/10.72	0.04/5.53

* OR = Odds Ratio;

† Log = Logistic;

‡ M = Median;

§ SD = Standard deviation;

|| CCI = Charlson Comorbidity Index;

¶ ERRD = Estimated Relative Risk of Death;

** Student's t test;

†† Chi-square test;

‡‡ Mann-Whitney's U test;

§§ Fisher's test

## Discussion

This study sought to analyze the variables that condition FD at three moments. It was observed that the demographic and clinical variables have a more significant impact between baseline and discharge, compared to other periods under analysis (t1 and t2). It was identified that the “advanced age”, “hospitalization in the last year”, “*delirium*”, and presenting “risk of decline” variables were transversal predictors to these moments. These data had already been identified in a systematic review study^(^ 5 ^)^, reinforcing the importance of incorporating in the initial assessment of the patients issues related to previous hospitalizations, to *delirium*, and to the assessment of the risk of functional decline.

Older adults with functional decline at t0 had more geriatric conditions (sight problems, risk of PI, cognitive changes, depression, *delirium*), did not have a partner, were previously hospitalized, and were restrained during hospitalization. These conditions are associated with worse health outcomes, namely FD during hospitalization^(^ 3 ^,^ 7 ^)^. This study is eloquent in the predictive value of *delirium* and of FD. It was observed that *delirium* at admission affects the functional trajectory between baseline and discharge and, on the other hand, *delirium* diagnosed during hospitalization affects the functional trajectory between discharge and follow-up and between baseline and follow-up. This result confirms that *delirium* is related to a worse functional recovery, as well as to longer hospitalization periods^(^ 35 ^-^ 37 ^)^. The assessment of *delirium* is not a systematic practice in Portugal and its recognition among nurses is low^(^ 38 ^)^. Likewise, the performance of a cognitive follow-up at the time of admission, which is also infrequent, must be considered as the first intervention in its prevention. However, the sample of patients with *delirium* is small (test power), which limits the accuracy of the results. On the other hand, the exclusion criteria may have discarded patients with more predisposing factors, therefore with a higher risk of developing *delirium*. Additionally, the assessment of *delirium* at admission occurred at a single moment and in the morning period. Future studies should consider its assessment at various times of the day and characterize it as to its onset and subtype.

The influence of previous hospitalizations is a relevant data and has already been identified in another study^(^ 3 ^)^. The data on unscheduled hospitalization (occurring within a period of 30 days after discharge) report a rate of 4.7%, being more significant in the age group of older adults^(^ 39 ^)^. Considering that 41.6% of the individuals do not recover their baseline, we may be facing a continuous cascade of functional loss between each hospitalization.

Another significant predictor was restraint^(^ 40 ^-^ 41 ^)^. The use of restraints is worsened during hospitalization and is linked to other predictors^(^ 41 ^)^, such as cognitive deficit, a predisposing factor for *delirium* which, in turn, can predetermine the use of restraints. This hospital practice is more frequent in older adults, who are already vulnerable to the adverse events resulting from restraints (e.g., incontinence, isolation, pressure injury, infection, *delirium*, and death due to aspiration)^(^ 41 ^-^ 42 ^)^. The use of alternative measures to the restraints must be considered in the context of Portuguese care. However, it will only be possible to reduce/eliminate the use of restraints by intervening in the causes, namely in the lack of human and material resources, in the insufficient training of the professionals in the management of behavioral and psychological symptoms^(^ 37 ^)^.

Older adults have a higher risk of functional decline at t1 and t2. The reduction in the functional reserve and in the resistance to balance-disturbing events can be an explanation. In this study, the mean age was 83 years old, which can represent a reduced functional reserve and deficits in multiple physiological systems, encompassing aspects such as decreased muscle mass, strength, resistance and balance, with lower activity capacity. Advanced age increases by 25% the prevalence of the frailty syndrome in individuals aged 80 and over^(^ 43 ^)^. Faced with a stressful event*,* such as hospitalization, frail older adults are at risk of severe deterioration in their physical and psychological well-being^(^ 44 ^-^ 45 ^)^, which can contribute to a more pronounced decline in these patients.

It was observed that the diagnosis is a predictor of functional decline after hospital discharge, in line with other studies^(^ 3 ^,^ 7 ^,^ 46 ^)^. This research analyzed the pathologies by two groups (infection and non-infection diagnoses). The group of pathologies classified as “non-infection” includes a wide range of diseases, reason why the analysis by groups of pathologies alone would be relevant to understand their predictive value in decline. The complexity of many of these pathologies, such as oncological disease, heart failure, chronic kidney injury, and chronic obstructive pulmonary disease, can result in greater functional limitations for the older adults, not only during hospitalization but also on their return home. The management of more complex signs, symptoms, and therapeutic regimens after discharge can increase the risk of adverse events, side effects and drug iatrogenesis. All these factors can contribute to the more pronounced functional decline between discharge and follow-up. These data reinforce the importance of the admission diagnosis in the decline after discharge, so that programs for the management of chronic disease and an articulation with resources in the community can be understood as strategies capable of mitigating functional decline in this group of patients with diagnoses associated to more chronic events.

Regarding the limitations of the study, we cite the following: the small sample size (n=101), which limits the power of the statistical tests to detect relevant associations in the analysis of the predictors of functional decline; the self-report of functional capacity, referring that the patients and/or caregivers can under- or over-estimate these values. A physical performance measure would allow obtaining more accurate data of functionality. As a third limitation, the non-inclusion of variables such as the number of falls in the last year, mobility of the patients in the hospital, socioeconomic status, and medication introduced during hospitalization. The inclusion of these variables could improve the variance explained by the models. Future studies should consider them in data collection. Although the missings of the study are not very significant, it is suggested to attribute these data using statistical algorithms (e.g., single linear regression method), in order to increase the number of cases in the logistic regression. The fourth limitation consisted in not assessing functional capacity at admission. Future studies should consider this assessment in order to determine the impact of acute diseases on pre-hospitalization. This data would be relevant to clarify the effect of the disease on the functional trajectory. Although the impact of an acute disease may be significant, the functional trajectory can be independent of it^(^ 30 ^)^, with hospitalization playing a very significant role^(^ 15 ^)^. The fifth limitation is that the assessment during hospitalization took place at a single moment. Extending this assessment to more moments could allow for the identification of a greater number of predictors. Finally, these results must be generalized with caution, taking into account the specific context (internal medicine services) in a university hospital. Multicenter studies would be decisive to determine the predictors of functional decline among hospitalized older adults in Portugal.

This study allows evidencing the factors that most contributed to functional decline in the older adults hospitalized in internal medicine services, with the following standing out: age, hospitalization in the last year, cognitive deficit, restraint, and *delirium*. Some of these predictors stem from the health-disease process; however, others can be associated with the hospital practice.

## Conclusion

This study showed that the transversal predictors to FD were as follows: advanced age, hospitalization in the last year, *delirium*, and having an assessment of risk of decline. During hospitalization, the most significant predictors of FD were the following: previous hospitalization, access to social support, cognitive deficit, mechanical restraint, and not having a partner. Between discharge and follow-up, the main predictors of FD were age and medical diagnosis. Finally, between baseline and follow-up, advanced age, *delirium*, and the risk of FD were the most relevant predictors. Implementing targeted interventions with specific guidelines for these predictors, especially those associated with the hospital practice, such as the use of restraints and *delirium*, will certainly contribute to preventing FD in hospitalized older adults.
